# Experimental investigations of the human oesophagus: anisotropic properties of the embalmed mucosa–submucosa layer under large deformation

**DOI:** 10.1007/s10237-022-01613-1

**Published:** 2022-08-28

**Authors:** Ciara Durcan, Mokarram Hossain, Grégory Chagnon, Djordje Perić, Georges Karam, Lara Bsiesy, Edouard Girard

**Affiliations:** 1 Zienkiewicz Centre for Computational Engineering, Faculty of Science and Engineering, Swansea University, Swansea, SA1 8EN UK; 2 Université Grenoble Alpes, CNRS, UMR 5525, VetAgro Sup, Grenoble INP, TIMC, 38000 Grenoble, France; 3 Laboratoire d’Anatomie des Alpes Françaises, Université Grenoble Alpes, Grenoble, France

**Keywords:** Human oesophagus, Cyclic testing, Visco-hyperelasticity, Anisotropy, Stress-softening, Damage mechanics

## Abstract

Mechanical characterisation of the layer-specific, viscoelastic properties of the human oesophagus is crucial in furthering the development of devices emerging in the field, such as robotic endoscopic biopsy devices, as well as in enhancing the realism, and therefore effectiveness, of surgical simulations. In this study, the viscoelastic and stress-softening behaviour of the passive human oesophagus was investigated through *ex vivo* cyclic mechanical tests. Due to restrictions placed on the laboratory as a result of COVID-19, only oesophagi from cadavers fixed in formalin were allowed for testing. Three oesophagi in total were separated into their two main layers and the mucosa–submucosa layer was investigated. A series of uniaxial tensile tests were conducted in the form of increasing stretch level cyclic tests at two different strain rates: 1% s$^{-1}$ and 10% s$^{-1}$. Rectangular samples in both the longitudinal and circumferential directions were tested to observe any anisotropy. Histological analysis was also performed through a variety of staining methods. Overall, the longitudinal direction was found to be much stiffer than the circumferential direction. Stress-softening was observed in both directions, as well as permanent set and hysteresis. Strain rate-dependent behaviour was also apparent in the two directions, with an increase in strain rate resulting in an increase in stiffness. This strain rate dependency was more pronounced in the longitudinal direction than the circumferential direction. Finally, the results were discussed in regard to the histological content of the layer, and the behaviour was modelled and validated using a visco-hyperelastic matrix-fibre model.

## Introduction

The oesophagus is a mechanical organ that transports food, in the form of a fluid bolus, from the pharynx to the stomach through a process called peristalsis (Payan and Ohayon 2017). The hollow muscular tube goes through a series of cyclic contractions in the longitudinal and circumferential directions, as well as circumferential distention, during primary and secondary peristalsis (Mir et al. 2016). In addition, boluses of different sizes pass through the oesophagus. Therefore, it is of interest to study the tissue’s material behaviour over repeated cycles and different stretch levels. Furthermore, the mechanical characterisation of the organ has a wide variety of applications, including within medical device design, surgical simulations, and tissue engineering (Lin et al. 2020; Yim and Sitti 2011; Arakelian et al. 2018; Sommer et al. 2013). Recent developments in the field of soft robotics have led to endoscopy devices able to perform biopsies within the gastrointestinal (GI) tract; piercing between its layers to extract samples suspected to be submucosal tumours (Son et al. 2020; Alsunaydih and Yuce 2021; Simi et al. 2013; Hoang et al. 2020). However, without adequate mechanical characterisation and modelling of the individual layers of the human GI tract, medically-relevant computational models cannot be developed to aid in the design of such devices. The potential benefits afforded by these models are undeniable, resulting in savings of resources such as time, materials, and biological test specimens. Moreover, the characterisation of the viscoelastic properties of the human oesophagus will benefit surgical simulations in increasing their realism by producing a training experience that is both stress/strain-dependent and time-dependent (Taylor et al. 2009). Further to this, in tissue engineering, knowledge of the mechanical behaviour of native oesophageal tissue can be used to compare against that of the grown tissue to ensure that the later’s material properties are sufficiently close to the former’s.

The oesophagus is the only visceral organ that can be easily separated into distinct layers (Payan and Ohayon 2017). This allows for the organ to be treated as a multi-layered composite material with differing mechanical properties within each layer. Experimentation carried out on the human oesophagus concludes that the intact wall behaves as a non-linear material (Vanags et al. 2003; Egorov et al. 2002), while also displaying anisotropic properties with greater stiffness in the longitudinal direction than the circumferential direction (Vanags et al. 2003). These findings are in agreement with similar studies on animal oesophagi (Sommer et al. 2013; Stavropoulou et al. 2012; Yang et al. 2004; Zhao et al. 2007; Gregersen et al. 2008). The previous studies investigating the human oesophagus, however, only explored its hyperelastic behaviour and did not consider its layer-dependent properties.

Investigations into the cyclic behaviour of a wide range of soft tissues, from skin to the aorta to the brain, have been conducted on both animal tissues (Emery et al. 1997; Gregersen et al. 1998; Giles et al. 2007; Van Loocke et al. 2009; Jayyosi et al. 2018; Remache et al. 2018), including the oesophagus (Yang et al. 2006a, b; Saxena et al. 2021), and human tissues (Peña et al. 2011; Rubod et al. 2012; Weisbecker et al. 2012; Fereidoonnezhad et al. 2016; Budday et al. 2017; Masri et al. 2018; Anttila et al. 2019). Currently, studies investigating the softening of the oesophagus have only been carried out using animal tissue (Liao et al. 2009; Jiang et al. 2014, 2017, 2019). Liao et al. (2009) used guinea pig oesophagi to investigate the predominant mode of softening, whether either through stress-softening (known as the Mullins effect for polymers), in which the previous maximum strain affects the loss of stiffness, or softening due to the material's viscoelastic behaviour, wherein the time-dependent properties contribute to the loss of stiffness seen. It was concluded that both modes had an influence, however that stress-softening was the predominant mode, attributing to 90% of the stiffness loss. This is supported by the earlier findings of Gregersen et al. (1998) who conducted similar studies on the guinea pig small intestine. Liao et al. (2009) also determined the softening behaviour to be anisotropic, with greater softening effects seen in the circumferential direction than the longitudinal direction for the intact oesophageal wall. Further to this, Jiang et al. (2014) performed experimentation on rat oesophagi, looking at the effect of stress-softening on its passive stiffness, and investigating whether active muscle contraction had any effect on this. They found that the softening of the oesophageal wall could be reversed through muscular contractions induced by potassium chloride (KCl). This finding is very interesting in terms of the oesophagus’ physiological function wherein the organ passively distends due to the entering of the bolus (Paterson 2006), subsequently reducing the stiffness of the oesophageal wall. This distension triggers peristalsis through the mechano-sensory response and then, via the peristaltic muscular contractions, the stress-softening is reversed and the stiffness of the wall returns to the degree at which it was before the bolus passed. This was proposed by Jiang et al. (2014) to be some form of ‘self-protection’ for the oesophagus. The authors then went on to study this phenomenon in each of the two main layers of the rat oesophagus (Jiang et al. 2017), and found that the passive stiffness and energy loss were reversible in both layers upon KCl activation.

Currently, outside of the authors’ own work (Durcan et al. 2022) and to the best of their knowledge, there are no experimental studies regarding the layer-dependent, anisotropic mechanical properties of the human oesophagus, particularly concerning the organ’s viscoelastic response; the establishment of which has a variety of applications within medicine and engineering. Although fresh cadavers are preferential when characterising soft tissues, due to restrictions placed on the laboratory caused by the COVID-19 pandemic, fresh cadavers were not allowed for dissection. Therefore, this paper aims to provide new insight into the layer-specific behaviour of the human oesophagus through experimentation of the mucosa–submucosa layer extracted from cadavers fixed in formalin. In the recent work by Durcan et al. (2022), the embalmed muscularis propria layer was characterised. In the current study, the mucosa–submucosa is investigated through uniaxial tensile tests conducted in two different directions. These experiments were performed in the form of increasing stretch level cyclic tests to observe a range of mechanical behaviour; the experimental procedure of which has been outlined in Sect. 2. In Sect. 3, the results of the mechanical tests and histological analysis are presented. The mechanical behaviour of the mucosa–submucosa layer is simulated in Sect. 4 using an anisotropic, visco-hyperelastic matrix-fibre model. The results and modelling, with regard to the histological content of the layer, are discussed in Sect. 5. Finally, Sect. 6 summarises the findings of the study and outlines the plans for future work.

## Experimental methods

### Anatomical description of the oesophagus

**Fig. 1:**
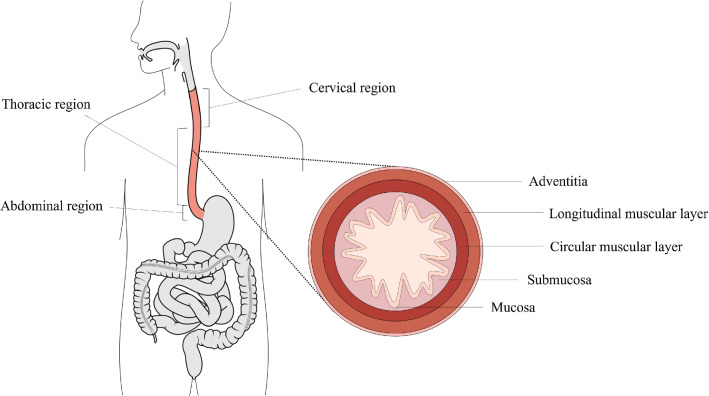
Diagram showing the position of the oesophagus in relation to the rest of the body (modified from Remesz (*wikimedia commons*) and a transverse segment of the oesophagus showing its histological layers

The oesophagus is an organ of the digestive system whose role is primarily mechanical in propelling food from the pharynx to the stomach. The organ is situated in the thoracic cavity and is divided into three regions; the cervical, thoracic and abdominal regions (Ferhatoglu and Kıvılcım 2017), as seen in Fig. 1. The cervical region is 5–6 cm in length and comprises the proximal end of the organ. The thoracic region is the largest of the three, approximately 17 cm in length, and is the middle section of the organ. The final region is the abdominal region which is situated adjacently to the stomach. This region is the smallest being 1–2.5 cm in length.

The gastrointestinal organ is made up of several distinct histological layers, as seen in Fig. 1, which, most notably, can be separated into two main layers post-explantation; the mucosa–submucosa layer and the muscularis propria. The mucosa is the innermost layer of the oesophagus and is comprised of three separate layers itself; the epithelium, the lamina propria and the lamina muscularis mucosae (Ferhatoglu and Kıvılcım 2017). The layer adjacent to this is the submucosa which consists of dense, irregular connective tissue made up of elastin and collagen fibres, and contains lymphatics, veins and the submucosal plexus. The muscularis propria layer comprises an innermost layer of circular muscle fibres, followed by a layer of longitudinal muscle fibres, and then finally the outermost layer, the adventitia (Ferhatoglu and Kıvılcım 2017). Between the muscular layers exists a thin layer of connective tissue which contains the majority of collagen and elastin within this layer.

**Fig. 2:**
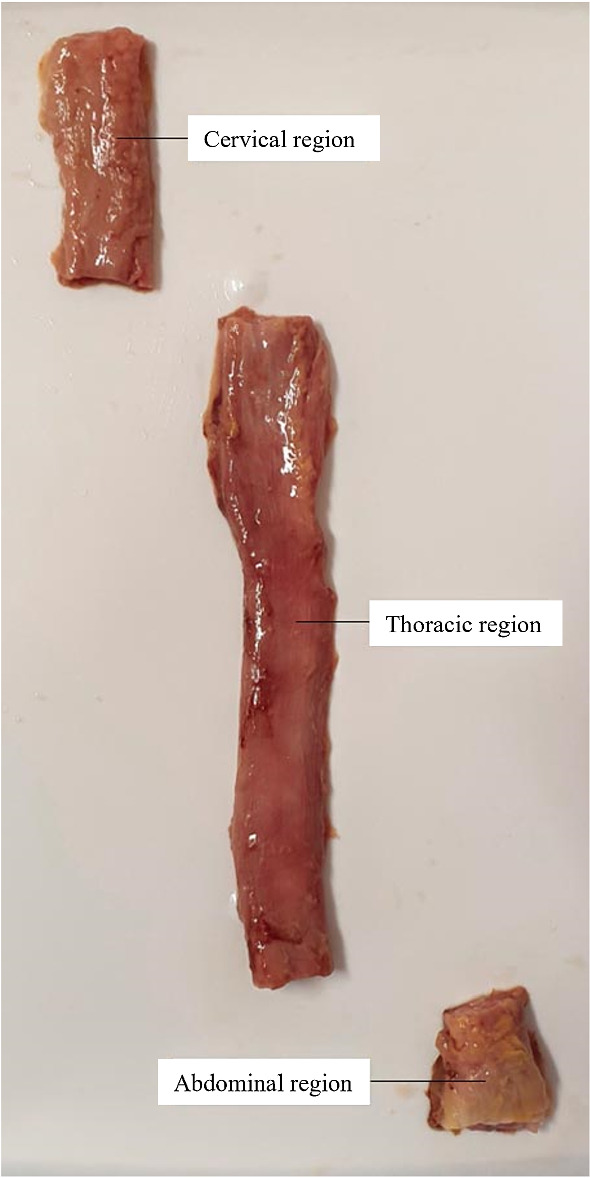
A human oesophagus divided into its three main regions (cervical, thoracic, and abdominal)

### Sample extraction

Dissection was performed at the Laboratoire d’Anatomie Des Alpes Françaises, Grenoble, France in order to extract the three whole human oesophagi for testing. The same procedure was repeated for each oesophagus. Due to the COVID-19 restrictions, wherein fresh cadavers were not allowed for dissection, the organs were retrieved from embalmed cadavers. Cadavers were embalmed using a formalin solution (ARTHYL) which was injected into the carotid artery and subsequently drained from the jugular vein. After embalming and prior to dissection, the cadavers were preserved in a $4^\circ$ C refrigerated room.

Oesophagectomy was realised through a midline sterno-laparotomy. The left triangular ligament was incised and the liver left lobe was retracted. The gastrohepatic ligament was incised, and an upper gastric section was realised. A phrenotomy was performed until the hiatus, the phreno-oesophageal ligament was incised and the oesophagus was circumferentially dissected. The thoracic oesophagus was released by a left approach after having reclined the left lung and sectioned the azygos vein. Finally, a subglottic oesophageal section was performed after a left cervical approach. The study was performed in compliance with French regulations on postmortem testing, and the protocol was approved by a local scientific committee of Université Grenoble Alpes.

### Histology

Prior to layer separation, samples from the intact organ of Cadaver 1 were reserved for histological analysis in both the transversal and longitudinal planes. Once the layers of this organ were separated, a sample was then obtained in the coronal plane from the mucosa–submucosa layer. The samples were all conserved in formaldehyde, fixed first in formalin 10% for 24 h at $4^\circ$ C, and then embedded in paraffin according to usual protocol (Canene-Adams 2013). Sections were realised $3\upmu \,\mathrm{m}$ in size with a microtome Leica RM 2245 (Wetzlar, Germany). The slides were stained with Sirius Red, Haematoxylin Eosin Saffron (HES) and Orecin. Sirius Red highlights the muscular fibres and all types of collagen, HES shows the nucleic acids and connective tissue (amongst other collagen), and Orecin stains the elastin fibres.

### Sample preparation

**Fig. 3:**
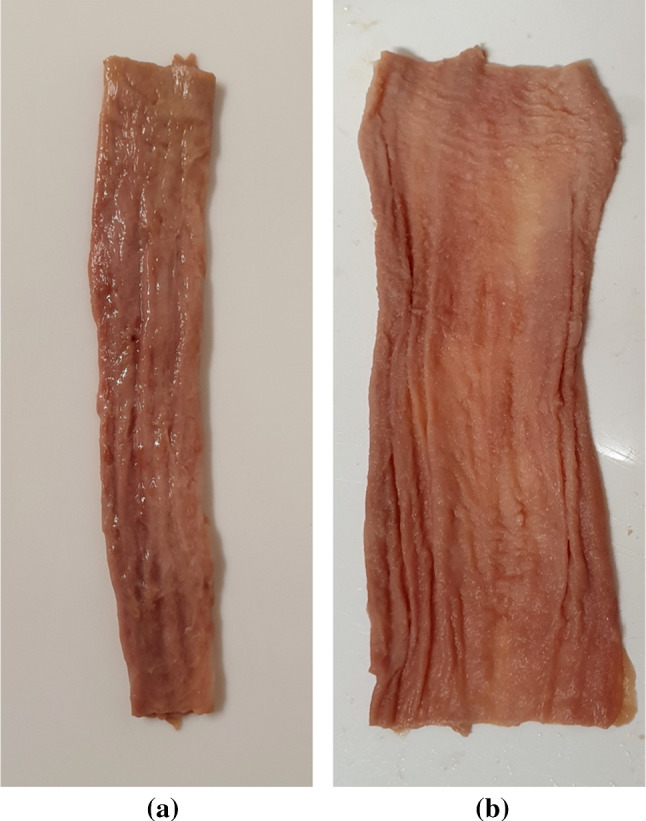
Section of the thoracic mucosa–submucosa in its original tubular form after layer separation (**a**), and the same section unravelled (**b**)

The oesophagi from Cadavers 1, 2 and 3 were approximately 25 cm, 26 cm and 22 cm in size, respectively, and, after explantation, were all cleaned in preparation for testing by removing any excess connective tissue with a scalpel. The oesophagi were then cut into their three separate regions (cervical, thoracic, and abdominal), as seen in Fig. 2, by cutting along the circumferential direction. Only the thoracic region was used for testing as it constitutes the majority of the tissue, thus reducing the effect of potential region-dependent properties on the results. In order to separate the layers of the thoracic region, first a cut, administered only to the muscular layer, was made along its longitudinal length. The opening created was then used to carefully deliver a series of small cuts to the connective tissue binding the mucosa–submucosa and the muscularis propria together. An example of the fully separated mucosa–submucosa can be seen in Fig. 3a. The mucosa–submucosa layers from each cadaver were then unravelled, as demonstrated in Fig. 3b, in preparation for the samples to be cut. The layers were flattened and rectangular samples approximately 22.00 mm $\times$ 4.10 mm (length $\times$ width) in size were cut in both the longitudinal and circumferential directions. This process often proved difficult due to the very soft and sticky nature of the layer. The testing was completed within 5 days of explantation, during which the tissue was stored in physiological saline solution (0.9% NaCl) in a $4^\circ$ C refrigerator. Before testing, the mucosa–submucosa was brought to ambient temperature, and new samples were cut each day. The samples were kept moist with saline solution between tests. To note, when analysing the results, no correlation was found to suggest that the length of time between explantation and testing had an influence on the mechanical properties of the samples.

### Experimental setup

**Fig. 4:**
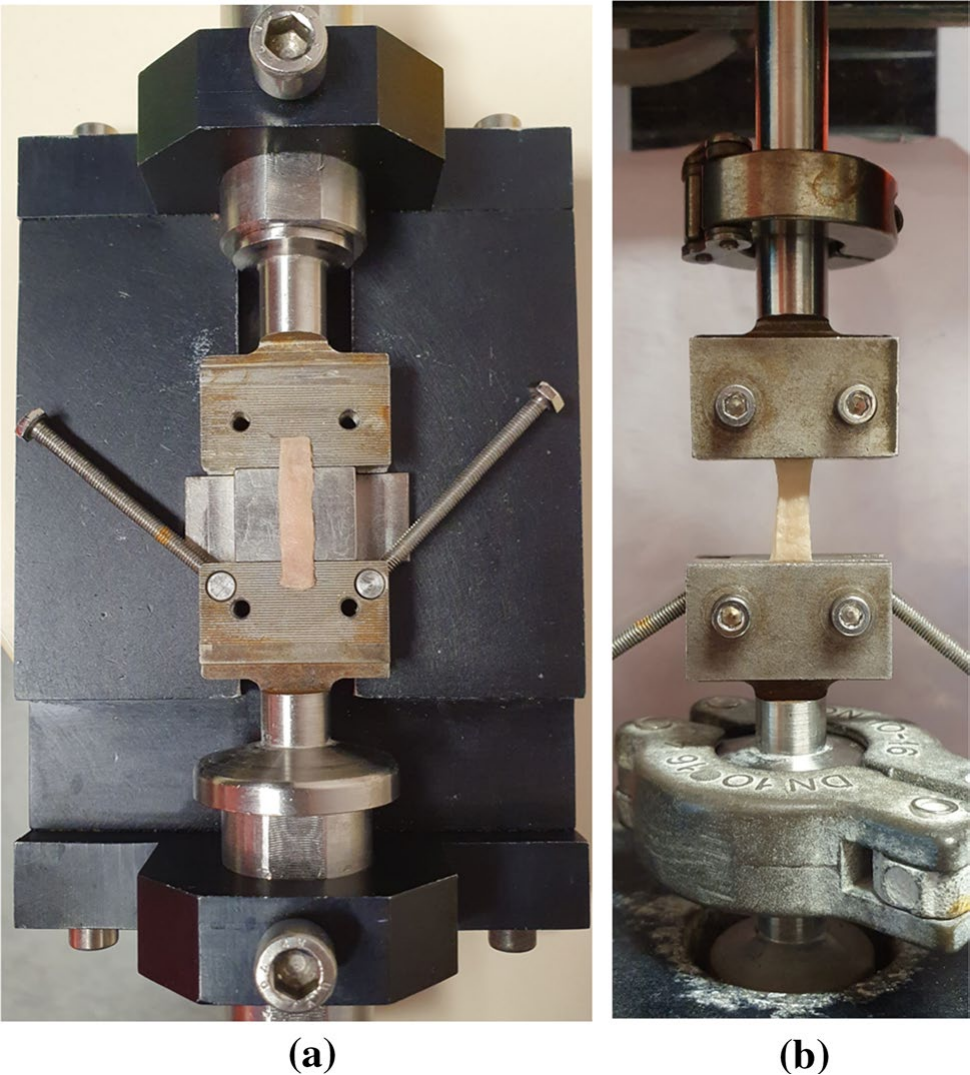
**a** Rectangular sample being loaded between the grips. **b** Sample loaded in the machine

To load the sample within the machine, a specially designed device was used, as seen in Fig. 4a. Firstly, the sample was aligned as centrally as possible upon the lower grips. This step took some time due to the sticky and delicate composition of the sample. Next, the upper part of the grips were added and the screws tightened using a torque limiter set at 0.5 Nm to ensure consistency and to prevent the sample from slipping during testing. The long screws either side of the support were then tightened, creating an assembly in which the positioned soft tissue sample could be moved. The assembly was then attached to a 25 N load cell in an MTS Criterion (model C41) traction machine. Once setup in the machine, the long screws of the assembly were untightened and the back support was removed, leaving the sample loaded within the machine as shown in Fig. 4b. Adjustments in the crosshead were made to ensure the samples were not buckling, with any amendments being added to their previously recorded initial length. At this point, the width and thickness of the samples were measured at three different points along their length using a calliper, and an average was taken. The deformation of the samples was computed from measurements taken by an extensometer in the traction machine which determined the displacement of the crosshead. The strain was then calculated from the grip-to-grip length of the sample, in which the length-to-width ratio was approximately 4:1, in line with the ASTM standards for uniaxial tensile testing (ASTM 2013). The machine was controlled, and the test parameters inputted, using the MTS TestSuite software.

### Mechanical characterisations

The experimental strain is expressed in terms of stretch, $\lambda$, which relates to nominal strain by $\varepsilon =\lambda - 1$. Stretch is defined as $\lambda = \frac{l}{l_0}$, where *l* and $l_0$ are the current and initial lengths of the specimen, respectively. The strain rates are expressed in units of percentage deformation per second (% s$^{-1}$). The stress is expressed as nominal stress, i.e. the first Piola-Kirchhoff stress, which is defined as:

1 $$\begin{aligned} P = \frac{F}{A_0} \end{aligned}$$

where *F* is the applied force and $A_0$ is the original, undeformed cross-sectional area.

**Fig. 5:**
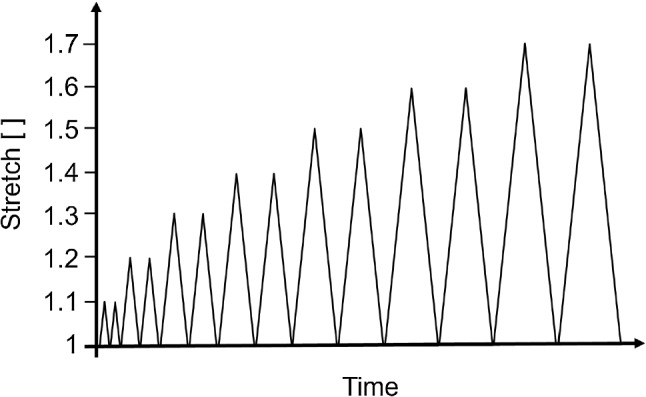
Stretch-time schematic of the mechanical test protocol

Cyclic tests consisting of a series of loading and unloading phases with increasing stretch levels were used to investigate the viscoelastic behaviour of the oesophageal mucosa–submucosa. In order to understand the effect of preconditioning, each stretch level cycle was repeated twice. Due to the limited number of human tissue specimens available, this form of test was chosen over a single cycle or a single deformation level cyclic test to be able to observe the most phenomena whilst testing the fewest samples. Stretch levels of 1.1, 1.2, 1.3, 1.4, 1.5, 1.6 and 1.7 were chosen; the stretch-time protocol of which can be seen in Fig. 5. If the samples underwent a clear rupture before reaching the final 1.7 stretch level, the test would be terminated. The stretch levels were based on the isobaric *in vivo* distension tests conducted by Takeda et al. (2002) who found the circumferential stretch of the oesophagus to be in the range of 1.15–1.70. The cyclic tests were conducted at two different strain rates, 1% s$^{-1}$ and 10% s$^{-1}$, to explore any rate-dependent behaviour of the tissue. Each cyclic test was performed 5–10 times per direction, per strain rate, per cadaver, and a new sample was used for each new test. To reiterate, no repeat tests were performed on the same sample; all tests were conducted until 1.7 stretch or until rupture. All experiments were carried out at ambient temperature and under a uniaxial tensile test condition.

## Results

### Histological analysis of the mucosa–submucosa layer of the human oesophagus

**Fig. 6:**
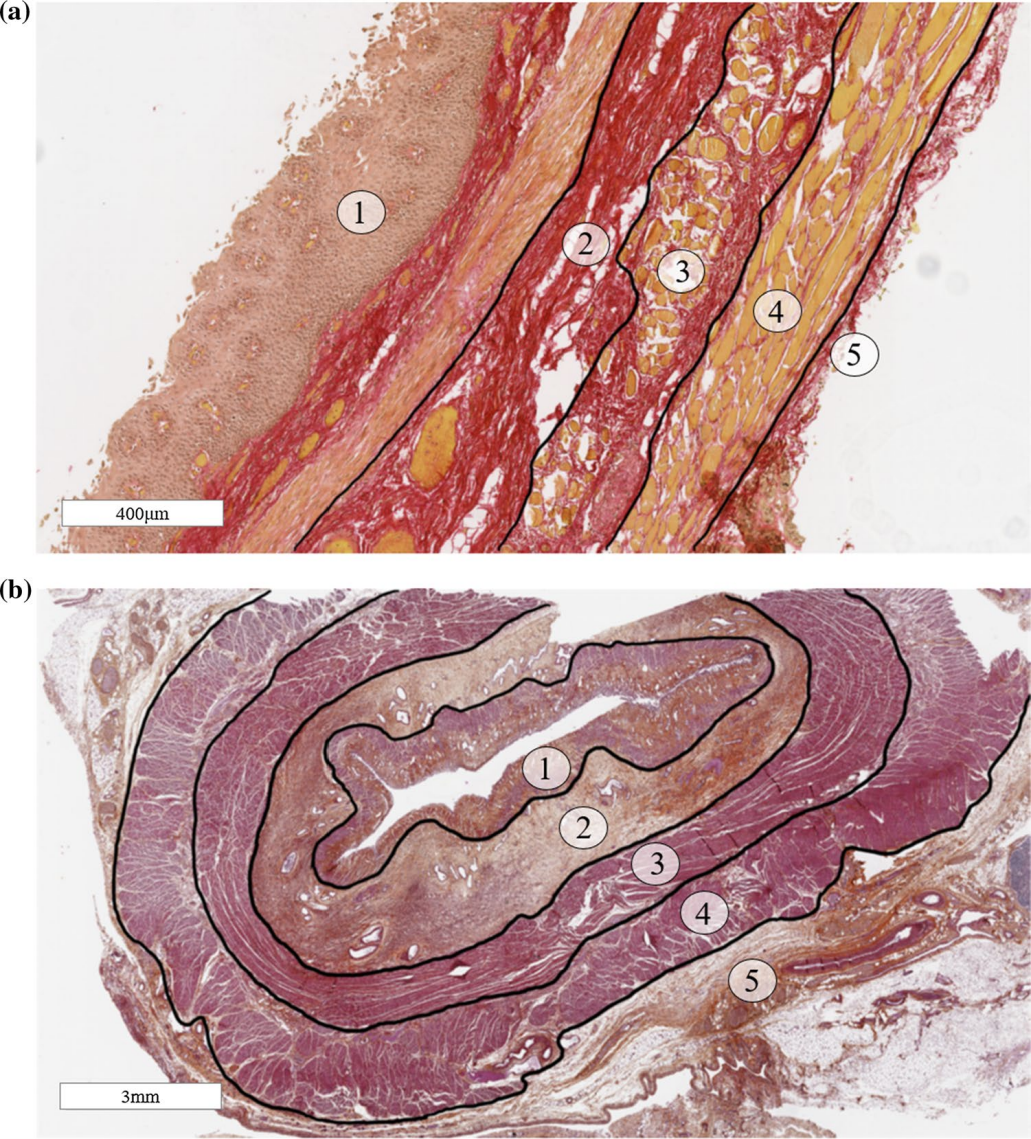
Sirius Red staining in the longitudinal plane (**a**) and Haematoxylin Eosin Saffron staining in the transversal plane (**b**) showing the mucosa (**1**), submucosa (**2**), the circular muscle fibres of the muscularis propria (**3**), the longitudinal muscle fibres of the muscularis propria (**4**) and the adventitia (**5**)

In the longitudinal plane, the layers of the oesophagus outlined in Sect. 2.1 are clearly visible, as seen in Fig. 6a. These layers are also evident in the transversal plane, as seen in Fig. 6b. The mucosa and the submucosa layers are richer in collagen and elastin fibres than the muscularis propria. The distribution of these fibres within the mucosa–submucosa have been summarised in Table 1. The mucosa contains more collagen fibres than elastin, with the collagen fibres being mainly oriented longitudinally, which is also the case for the elastin fibres of the layer. The muscle fibres of muscularis mucosae are longitudinally oriented as well. In the submucosa, there is more collagen than elastin. The collagen and elastin fibres do not have the same orientation throughout the thickness of this layer. In the inner part, the collagen and elastin fibres are oriented longitudinally in the direction of the oesophagus. In the outer part, close to the muscularis propria, the fibres are oriented transversely, following the muscle fibres of the adjacent inner circular muscular layer.

**Table 1:** Distribution of collagen and elastin in the mucosa–submucosa; +, low density; +++++, high density

Layer	Collagen	Elastin
Mucosa	+++++	+
Submucosa	+++++	++

### Demographics and variations in experimental samples

The oesophageal mucosa–submucosa of three cadavers was tested in total. The demographics of the three patients can be found in Table 2. Due to the variable nature of biological tissue, dimensions such as the thickness can vary, both inter- and intracadaver. The variation of dimensions across all samples is presented in Table 3.

**Table 2:** Patient demographics and time of embalming

Cadaver	Sex	Height (cm)	Weight (kg)	Age (years)	Time of embalming (days)
1	Female	165	75	88	24
2	Female	153	40	97	71
3	Female	140	40	101	40

**Table 3:** Mean ± population standard deviation of sample dimensions

	1% s$^{-1}$		10% s$^{-1}$	
	Width (mm)	Thickness (mm)	Width (mm)	Thickness (mm)
Longitudinal	4.05 ± 0.37	0.75 ± 0.16	4.06 ± 0.35	0.74 ± 0.12
Circumferential	4.12 ± 0.28	0.81 ± 0.17	4.27 ± 0.34	0.79 ± 0.12

### Presentation of the experimental results

Cyclic tests were conducted with two cycles per stretch level, and both cycles have been presented here. The majority of samples ruptured before reaching 70% strain, particularly the longitudinal samples; therefore, in the following graphs, only the full cycles have been presented. This means that if a sample ruptured during the first 1.2 stretch cycle, only the two 1.1 stretch cycles are presented.

### Reproducibility in the stress–strain data and statistical analysis

**Fig. 7:**
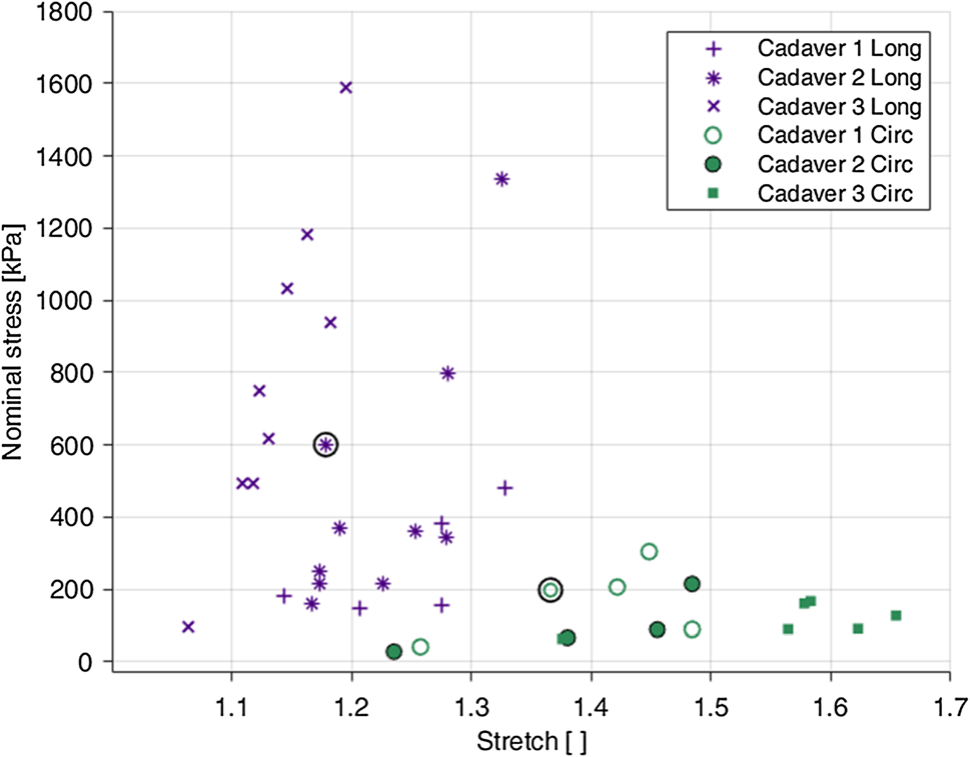
Rupture points of each test conducted at 10%$s^{-1}$, highlighting the dispersion between cadavers and the longitudinal and circumferential directions. The circled points show the rupture stress-stretch of the curves selected for analysis via statistical means

The total number of samples tested per test condition (i.e. a certain direction and strain-rate), per cadaver can be found in Table 4. When comparing the stress–strain data for a certain test condition, the results between samples were largely dispersed. This can be visualised in Fig. 7 which portrays the rupture stress-stretch of each sample tested at 10% s$^{-1}$, including a comparison between the two directions and the different cadaveric specimens. Rupture here is defined as irreversible macroscopic damage evident on the stress–strain curve as a sudden reduction in stress.

**Fig. 8:**
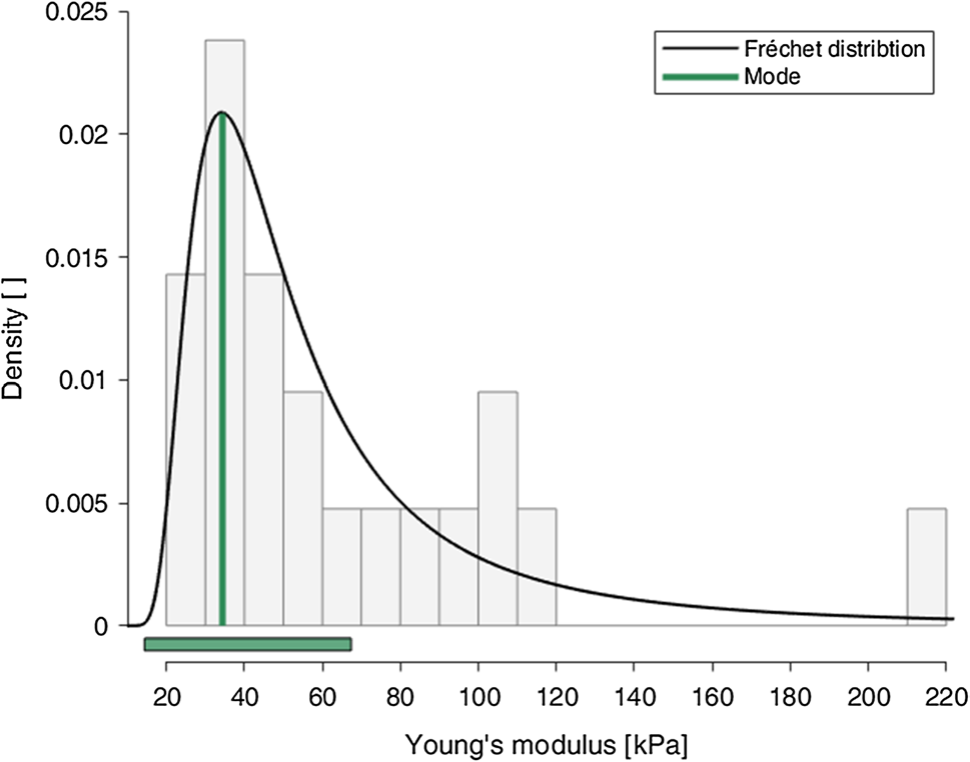
The combined histogram and probability distribution graph showing the dispersion of Young’s moduli for the 10%$s^{-1}$ circumferential experimental results across three cadavers displaying a significant ($p=0.975$) Fréchet distribution at $\alpha =0.05$ with a mode (range) of 34.4 (14.4–67.4). The vertical green line shows the mode of the probability distribution, while the horizontal green bar depicts the range

To obtain the most representative stress–strain data for analysis and constitutive modelling, a statistical approach was employed. For this, the distribution of Young’s moduli, *E*, for each test condition was evaluated and the most representative curve retrieved from this. Firstly, the Young’s modulus of each test was calculated by taking the gradient of the first loading curve of the first cycle from 1.00 to 1.01 stretch, with the modulus defined as:

2 $$\begin{aligned} E = \frac{P}{\varepsilon } \end{aligned}$$

where, *P* is the nominal stress and $\varepsilon$ is the nominal strain as described in Sect. 2.6. Next, a histogram with 20 bins was plotted for each test condition with density on the y-axis and Young’s modulus on the x-axis, as seen in Fig. 8. All test conditions presented a right-skewed histogram, highlighting a non-normal distribution of the moduli within the population. Therefore, several appropriate distributions were tested against a null hypothesis, including gamma distribution (Thom 1958), Fréchet distribution (Harlow 2002) and chi-squared distribution (Kissell and Poserina 2017). The null hypothesis for a specific test condition and distribution type was, “The Young’s modulus of the [specific] direction of the mucosa–submucosa layer of the embalmed human oesophagus tested at a strain rate of [specific]%$s^{-1}$ is distributed according to the [specific] distribution.”. The statistical tests were all performed using R Statistical Software and conducted at a significance level, $\alpha$, of $\alpha =0.05$, meaning that if $p<0.05$, the null hypothesis was rejected.

The null hypotheses were retained for the gamma and Fréchet distributions for all test conditions. However, the Fréchet distribution was chosen as it was the most appropriate for the application (Harlow 2002), with p-values of ($p=0.783$) for the 1%$s^{-1}$ circumferential results, ($p=0.247$) for the 1%$s^{-1}$ longitudinal results, ($p=0.975$) for 10%$s^{-1}$ circumferential results, as seen in Fig. 8, and ($p=0.899$) for the 10%$s^{-1}$ longitudinal results.

The mode of the Fréchet distribution presents the most likely Young’s modulus value within the population tested, while the range, in this circumstance, contains 70% of all Young’s moduli of the population. For the most representative behaviour of the mucosa–submucosa layer, the curve with the Young’s modulus closest to the mode of the Fréchet distribution for each test condition was chosen to conduct analysis and constitutive modelling. The mode (range) of the Young’s modulus for the 1%$s^{-1}$ circumferential results was 34.8 (21.2–47.5), 93.5 (25.8–331) for the 1%$s^{-1}$ longitudinal results, 34.4 (14.4–67.4) for the 10%$s^{-1}$ circumferential results, as seen in Fig. 8, and 122 (42.1–308) for the 10%$s^{-1}$ longitudinal results. The rupture points of the curves selected for analysis for each direction of the 10%$s^{-1}$ experimental results can be found circled in Fig. 7, the stress–strain cyclic data of which will be subsequently presented.

**Table 4:** Number of tests per direction, per strain rate, per cadaver

Direction	Strain rate	Cadaver	Tests	Total
Longitudinal		1	5	
	1%/s	2	10	*n* = 24
		3	9	
		1	5	
	10%/s	2	10	*n* = 24
		3	9	
Circumferential		1	5	
	1%/s	2	10	*n* = 23
		3	8	
		1	5	
	10%/s	2	10	*n* = 21
		3	6	

### Anisotropic response

**Fig. 9:**
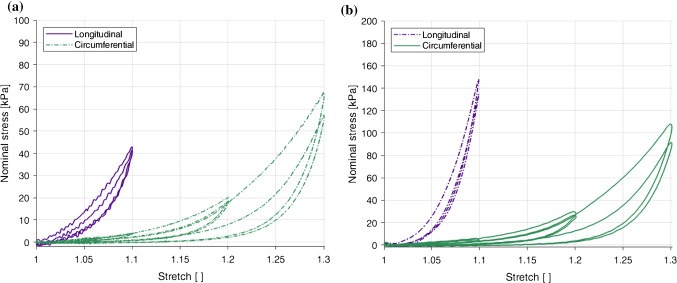
Effect of direction of loading on the results at 1% s$^{-1}$ (**a**) and 10% s$^{-1}$ (**b**) of the embalmed mucosa–submucosa layer

The embalmed mucosa–submucosa of the human oesophagus displayed anisotropic behaviour at both strain rates. The results of the 1% s$^{-1}$ tests can be seen in Fig. 9a and the results of the 10% s$^{-1}$ tests are depicted in Fig. 9b. The longitudinal direction was stiffer than the circumferential direction at both strain rates. It can also been seen that the longitudinal samples ruptured at a lower stretch level than the circumferential samples.

### Strain rate-dependent behaviour

**Fig. 10:**
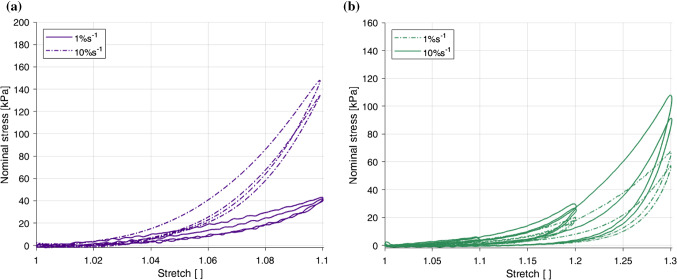
Effect of loading rate on the results in the longitudinal direction (**a**) and the circumferential direction (**b**) of the embalmed mucosa–submucosa layer

Figure 10 compares the two different strain rates for a single direction, with the longitudinal results in Fig. 10a and the circumferential results in Fig. 10b. A strain rate dependency was evident in both directions, with an increase in strain rate resulting in an increase in stiffness. A more pronounced dependency was observed for the longitudinal direction compared to the circumferential direction. While in the longitudinal direction the hysteresis does not seem to be significantly affected by the loading rate, the difference between the two loading curves for the 1.1 stretch level is greater at the higher strain rate. This implies that the stress-softening in this direction is greater with an increase in strain rate. Contrary to the longitudinal direction, hysteresis in the circumferential direction was found to be greater at 10% s$^{-1}$ than 1% s$^{-1}$.

### Permanent deformations

**Fig. 11:**
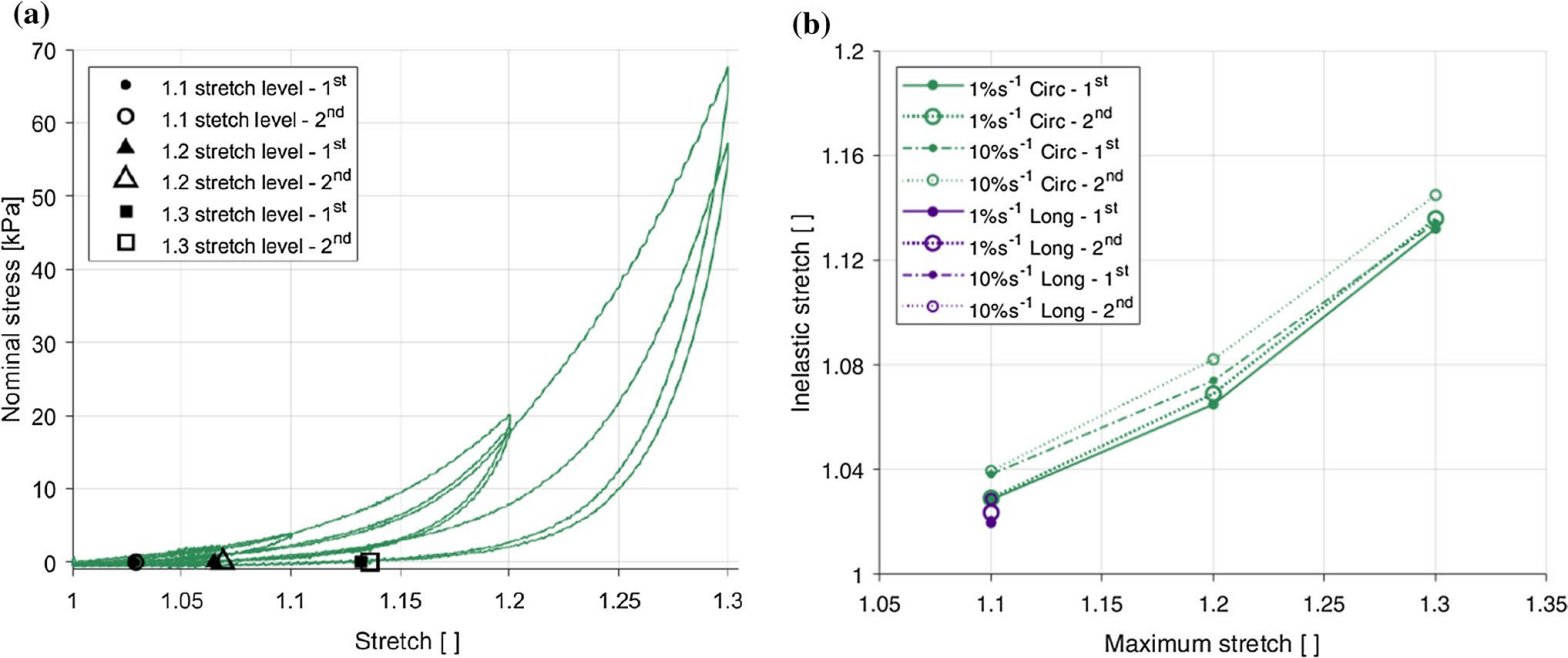
Markers showing the permanent set of the 1% s$^{-1}$ circumferential results for each stretch level and cycle (**a**), and permanent deformations in each loading direction corresponding to the maximum stretch of the previous cycle, for both the first and second cycles at both strain rates (1% s$^{-1}$ and 10% s$^{-1}$) (**b**)

Permanent deformations, also known as permanent set, refers to the residual, inelastic strains present in the tissue after the experimental load has been removed. Figure 11a presents the permanent stretch values for the 1% s$^{-1}$ circumferential results, i.e. the stretch of the unloading curves when $P=0$, and Fig. 11b shows the inelastic stretch with respect to the previous maximum stretch for each direction and each strain rate, including a comparison between the permanent set of the first cycle and the second cycle for a single stretch level. In the circumferential direction, permanent deformations were found to increase with an increase in stretch level for both strain rates and both cycles. For all directions and strain rates, the permanent deformations of the second cycle were greater than the first cycle for a single stretch level. The results suggest anisotropic behaviour, with slightly greater permanent set in the circumferential direction compared to the longitudinal direction. However, for this to be conclusive, smaller increments per stretch level are needed to observe more clearly the trend in the longitudinal direction. Strain rate effects can also be seen wherein the permanent set is greater at 10% s$^{-1}$ than 1% s$^{-1}$ for both directions.

## Constitutive modelling

In this section, we aim to model the mechanical behaviour of the embalmed mucosa–submucosa layer. Experimentally, we observed anisotropy, with distinct properties in each direction; viscoelastic behaviour, including hysteresis and a strain rate dependency in both directions; and damage, in the form of stress-softening and permanent deformations. A variety of approaches can be employed to capture the cyclic behaviour of soft tissues, including those based on continuum damage mechanics (CDM) (Maher et al. 2012; Balzani et al. 2012; Schmidt et al. 2014; Rodríguez et al. 2006), CDM combined with viscoelasticity (Mao et al. 2017; Wang and Chester 2018), and pseudo-elasticity (Fereidoonnezhad et al. 2016; Peña et al. 2009; Ehret and Itskov 2009). Here, we propose a unique formulation of an anisotropic, viscoelastic matrix-fibre model with an added stress-softening function to simulate the observed experimental behaviour.

### Anisotropic matrix-fibre model with damage

**Fig. 12:**
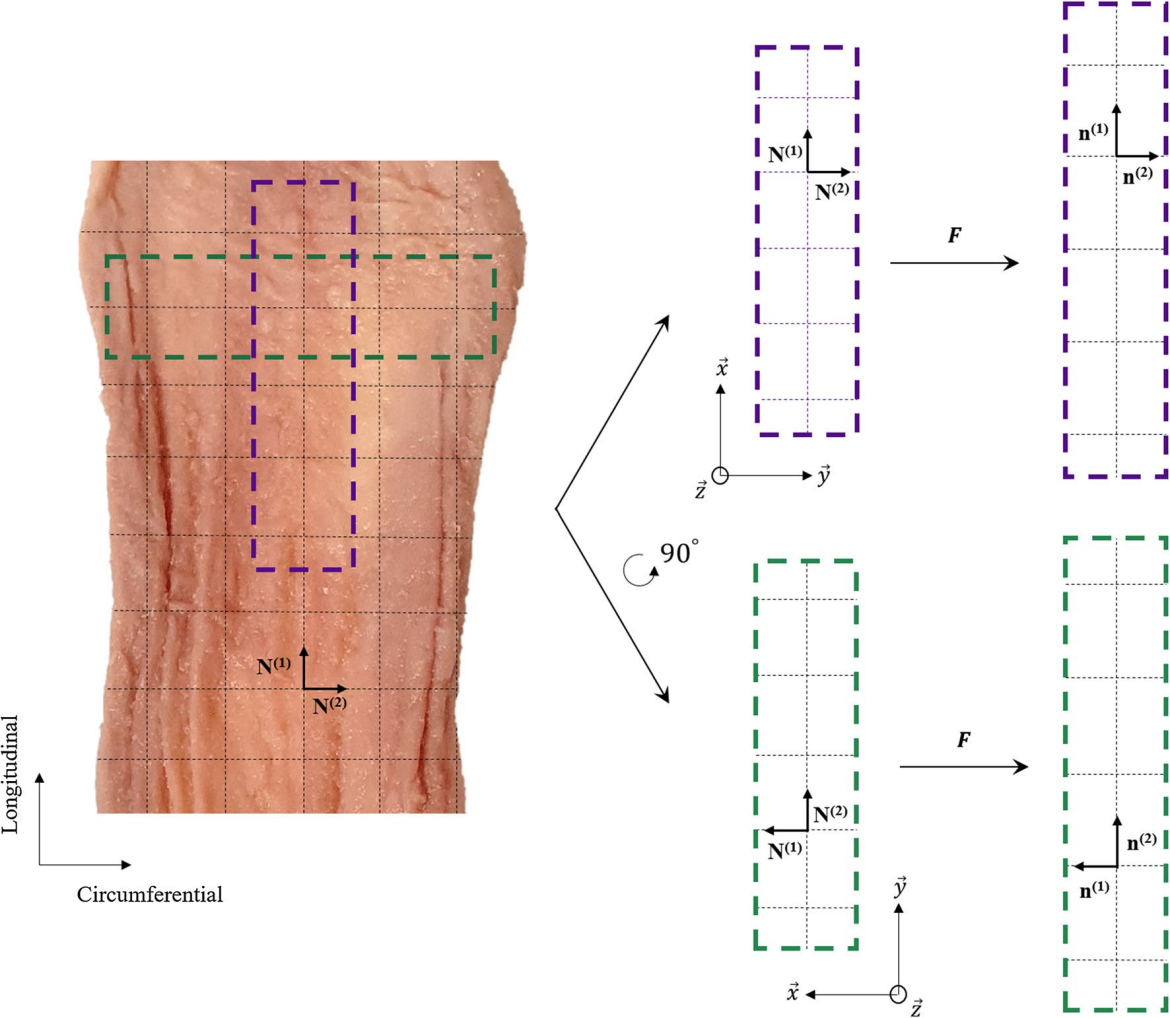
Drawing to illustrate the fibre orientation of the mucosa–submucosa layer of the human oesophagus based on the histological observations outlined in Sect. 3.1

First, the anisotropy is captured in the form of an orthotropic model. Within the matrix-fibre model, the matrix is assumed to be purely elastic and isotropic, with the anisotropy, damage and viscoelasticity originating from the collagen fibres and their predominant orientations (Yang et al. 2006a; Gautieri et al. 2012; Li et al. 2005). The histological analysis, outlined in Sect. 3.1, revealed that the collagen fibres reside mainly orthogonal to each other in the longitudinal and circumferential directions. This finding is logical when considering the embryology of the organ in which the oesophageal tube elongates inferiorly down the body (Esrefoglu et al. 2017). Therefore, contrary to similar animal studies (Sommer et al. 2013; Natali et al. 2009; Sokolis 2013), the collagen fibres of the mucosa–submucosa layer are captured by two families of fibres each running in the axes parallel to the experimental loading directions, i.e. perpendicularly to each other, as represented in Fig. 12. The stress can be defined either in terms of the reference configuration, the second Piola-Kirchhoff (PK) stress, or the deformed configuration, the Cauchy stress, and either will be used within this study depending on convenience. The second PK stress tensor for the orthotropic model for incompressible materials is described as:

3 $$\begin{aligned} \varvec{S} = -p\varvec{C}^{-1} + 2 \frac{\partial W_0}{\partial {I_1}}\varvec{I} + 2 \sum _{i=1,2}^{}{I_4^{\left( i\right) }\frac{\partial W_{\text {fibres}}^{\left( i\right) }}{\partial {I_4^{\left( i\right) }}}}\left[ \varvec{N}^{\left( i\right) }\otimes \varvec{N}^{\left( i\right) }\right] \end{aligned}$$

where, *p* is the hydrostatic pressure used to impose the incompressibility constraint, $\varvec{C}$ = $\varvec{F}^T\,\varvec{F}$ is the right Cauchy-Green tensor, $\varvec{F}$ is the deformation gradient tensor, $\varvec{I}$ is the identity tensor, $\varvec{N}^{\left( i\right) }$ is the direction of each set of fibres in the undeformed configuration, and $W_0$ and $W_{\text {fibres}}^{\left( i\right) }$ are the strain energy functions (SEFs) of the matrix and fibres, respectively, in which $I_1$ and $I^{(i)}_{4}$ are defined as:

4 $$\begin{aligned} I_1 = \text {tr}(\varvec{C}); \quad I^{(i)}_{4}= \varvec{C} :\left[ \varvec{N}^{(i)}\otimes \varvec{N}^{(i)}\right] . \end{aligned}$$

The Cauchy stress tensor for the orthotropic model for incompressible materials is:

5 $$\begin{aligned} \varvec{\sigma } = -p\varvec{I} + 2 \frac{\partial W_0}{\partial {I_1}}\varvec{b} + 2 \sum _{i=1,2}^{}{I_4^{\left( i\right) }\frac{\partial W_{\text {fibres}}^{\left( i\right) }}{\partial {I_4^{\left( i\right) }}}}\left[ \varvec{n}^{\left( i\right) }\otimes \varvec{n}^{\left( i\right) }\right] \end{aligned}$$

where, $\varvec{b}$ = $\varvec{F}\,\varvec{F}^T$ is the left Cauchy-Green tensor and $\varvec{n}^{\left( i\right) }$ is the direction of each set of fibres in the deformed configuration. The fibre orientation in the deformed state can be captured at any given time by $\varvec{n}^{(i)}$ = $\varvec{F}\,\varvec{N}^{(i)}$.

In addition to anisotropy, damage, in the form of stress-softening and permanent deformations, was observed within the experimental data. To capture this behaviour, a stress-softening evolution function, $\chi$, developed by Rebouah et al. (2013), which is also able to take into account the permanent set of a material, is employed. This function is described in the context of soft biological tissues by Rebouah and Chagnon (2014). As the matrix is considered to be purely elastic, the damage function is added to the fibre portion of the stress contribution as follows:

6 $$\begin{aligned} \varvec{\sigma } = -p\varvec{I} + 2 \frac{\partial W_0}{\partial {I_1}}\varvec{b} + 2 \sum _{i=1,2} {\chi ^{\left( i\right) }\left( I_4^{\left( i\right) },I_4^{\left( i\right) max}\right) I_4^{\left( i\right) }\frac{\partial W_{\text {fibres}}^{\left( i\right) }}{\partial {I_4^{\left( i\right) }}}}\left[ \varvec{n}^{\left( i\right) }\otimes \varvec{n}^{\left( i\right) }\right] . \end{aligned}$$

The stress-softening function added captures history-dependent behaviour by considering the difference between the current stretch and the previous maximum stretch, and is described as:

7 $$\begin{aligned} \chi ^{\left( i\right) }\left( I_4^{(i)},I_4^{(i)max}\right) =1-\eta _m^{\left( i\right) } \left[ \frac{I_4^{(i)max}-I_4^{(i)}}{I_4^{(i)max}-1}\right] ^{\beta ^{\left( i\right) }} \end{aligned}$$

where, $\eta _m$ and $\beta$ are dimensional parameters, and $I_4^{(i)max}$ is the maximum value of $I_4^{(i)}$ for each direction throughout the whole history of the material.

**Fig. 13:**
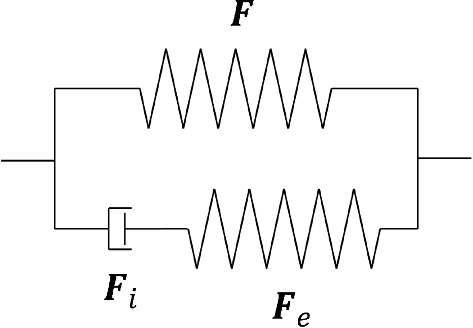
Rheological representation of the viscoelastic model

**Fig. 14:**
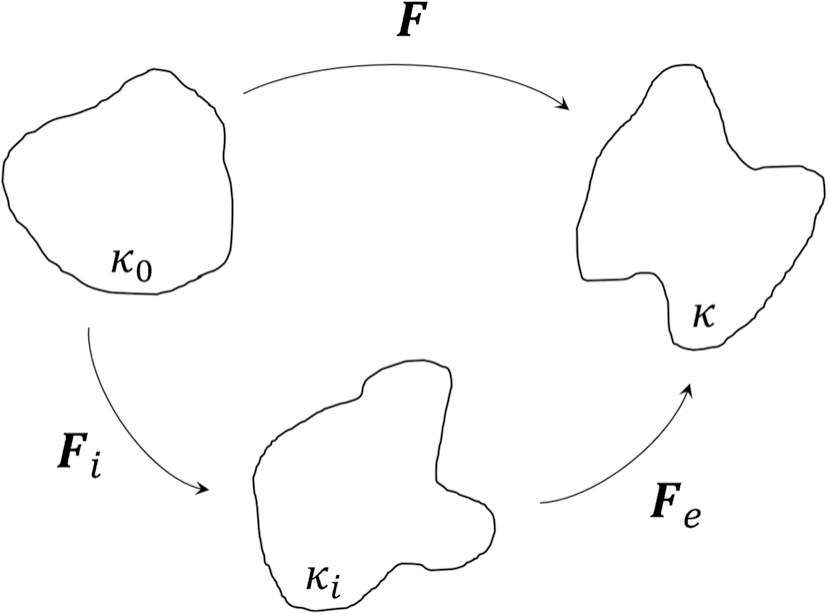
A decomposition of the deformation gradient tensor $\varvec{F}$, where $\kappa _0$ is the undeformed configuration, $\kappa _i$ is the intermediate configuration and $\kappa$ is the final configuration

Next, the viscoelasticity is considered and is also captured solely by the fibres. An internal variable-based model advocated by Petiteau et al. (2013) is employed, and can be represented using a spring-dashpot analogy. A schematic of the generalised Maxwell model can be seen in Fig.  13, where the deformation gradient tensor of the lower, inelastic branch can be described by a multiplicative decomposition into an elastic part, $\varvec{F}_e$, and an inelastic part, $\varvec{F}_i$, in which $\varvec{F} =\varvec{F}_e\varvec{F}_i$. Figure 14 shows a visual representation of this decomposition.

We assume that the viscoelasticity has no volumetric contributions, so the SEF of the fibres can be written as:

8 $$\begin{aligned} W_{\text {fibres}}(\varvec{F},\varvec{F}_e) = W_1(\varvec{F}) + W_2(\varvec{F}_e) \end{aligned}$$

where, $W_1$ is the SEF involved in the deformation between $\kappa _0$ and $\kappa$, and $W_2$ is associated with the deformation between $\kappa _i$ and $\kappa$. Therefore, the Cauchy stress simply becomes:

9 $$\begin{aligned} \varvec{\sigma } = -p\varvec{I} + 2 \frac{\partial W_0}{\partial {I_1}}\varvec{b} + 2 \sum _{i=1,2}^{}{\chi ^{\left( i\right) }\left[ I_4^{\left( i\right) }\frac{\partial W_1^{\left( i\right) }}{\partial {I_4^{\left( i\right) }}}\left[ \varvec{n}^{\left( i\right) }\otimes \varvec{n}^{\left( i\right) }\right] + I_{4,e}^{\left( i\right) }\frac{\partial W_2^{\left( i\right) }}{\partial {I_{4,e}^{\left( i\right) }}}\left[ \varvec{n}_e^{\left( i\right) }\otimes \varvec{n}_e^{\left( i\right) }\right] \right] } \end{aligned}$$

where,

10 $$\begin{aligned} I^{(i)}_{4,e}= \varvec{C}_e :\left[ \varvec{N}^{(i)}\otimes \varvec{N}^{(i)}\right] \end{aligned}$$

in which $\varvec{C}_e= \varvec{F}^T_e\,\varvec{F}_e$ is the right Cauchy-Green tensor related to the elastic deformation and $\varvec{n}_e^{(i)}$ = $\varvec{F}_e\,\varvec{N}^{(i)}$ is the orientation of the fibres in the elastically deformed state. The inelastic dashpot is expressed linearly with the viscosity parameter $\eta _0$. A thermodynamically-consistent evolution equation for the elastic deformation is described by Petiteau et al. (2013) and is defined for this case as:

11 $$\begin{aligned} \dot{\varvec{b}}_e=\varvec{L}\varvec{b}_e + \varvec{b}_e\varvec{L}^T-\frac{4}{\eta _0}\frac{\partial W_2}{\partial I_{4,e}}I_{4,e}\varvec{b}_e\left[ \left[ \varvec{n}_e\otimes \varvec{n}_e\right] -\frac{1}{3}I_{4,e}\varvec{I}\right] \end{aligned}$$

where, $\varvec{b}_e$ = $\varvec{F}_e\,\varvec{F}^T_e$ is the elastic deformation left Cauchy-Green tensor and $\varvec{L} = \dot{\varvec{F}}\varvec{F}^{-1}$ is the velocity gradient tensor.

### One-dimensional formulation of the model

Now the specific one-dimensional constitutive law will be formulated following the uniaxial tensile test condition. For this material, it is assumed that there are two main families of collagen fibres whose effect can be captured by their mean orientations in the undeformed configuration, $\varvec{N}^{\left( 1\right) }$ and $\varvec{N}^{\left( 2\right) }$, as seen in Fig. 12. For the longitudinal samples, the direction vectors are simply as follows:

12 $$\begin{aligned} \varvec{N}^{\left( 1\right) } = \begin{bmatrix} 1 & 0 & 0\\ \end{bmatrix}; \quad \varvec{N}^{\left( 2\right) } = \begin{bmatrix} 0 & 1 & 0\\ \end{bmatrix}. \end{aligned}$$

For uniaxial tension, the specimen is loaded in only one direction, i.e., $\lambda _x$ = $\lambda$ for the longitudinal samples, while the other two directions are unhindered. In this case, similarly to Holzapfel et al. (2000), it is assumed that the two families of fibres are active only in tension, i.e. $I^{(i)}_{4}\ge 1$ and $I^{(i)}_{4,e}\ge 1$. Therefore, for an incompressible material and due to the assumption of symmetry, the deformation gradient tensor for uniaxial tension can be written as:

13 $$\begin{aligned} \begin{bmatrix}\varvec{F}\end{bmatrix} = \begin{bmatrix} \lambda & 0 & 0 \\ 0 & \lambda ^{-\frac{1}{2}} & 0 \\ 0 & 0 & \lambda ^{-\frac{1}{2}} \end{bmatrix} \end{aligned}$$

where, $\lambda$ is the stretch as defined in Sect. 2.6. The matrix of the material is modelled using a neo-Hookean SEF as follows:

14 $$\begin{aligned} W_0 = c_1[I_1 - 3] \end{aligned}$$

where, $c_1$ is a stress-like material parameter. For the time-independent response of the fibres, a classic Holzapfel et al. (2000) SEF is used:

15 $$\begin{aligned} W_1^{\left( i\right) }=\frac{k_1^{\left( i\right) }}{2k_2^{\left( i\right) }}[e^{ k_2^{\left( i\right) } [I_4^{\left( i\right) }-1]^2}-1] \end{aligned}$$

where, $k_1 > 0$ is a stress-like material parameter and $k_2 > 0$ is a dimensionless parameter. For a comprehensive review of a variety of anisotropic, hyperelastic energy functions, readers are referred to Chagnon et al. (2015). An SEF from Kaliske (2000) is used to model the elastic deformation of the fibres (with $n=3$) and is defined as:

16 $$\begin{aligned} W_2^{\left( i\right) }=C_{2}^{\left( i\right) }[I_{4,e}^{\left( i\right) } - 1]^2 + C_{3}^{\left( i\right) }[I_{4,e}^{\left( i\right) } - 1] ^3 + C_{4}^{\left( i\right) }[I_{4,e}^{\left( i\right) } - 1] ^4 \end{aligned}$$

where, $C_{2}$, $C_{3}$ and $C_{4}$ are stress-like material parameters. The first PK tensor is related to the Cauchy stress by $\varvec{P} = J \varvec{\sigma }\,\varvec{F}^{-T}$. Solving for the unknown hydrostatic pressure, the one-dimensional first PK stress for the longitudinal direction in uniaxial tension becomes:

17 $$\begin{aligned} P^{\text {Long}} = 2 \frac{\partial W_0}{\partial {I_1}}[\lambda - \lambda ^{-2}] + 2\chi ^{\left( 1\right) }\lambda ^{-1}\left[ I_4^{\left( 1\right) }\frac{\partial W_1^{\left( 1\right) }}{\partial {I_4^{\left( 1\right) }}}\lambda ^2 + I_{4,e}^{\left( 1\right) }\frac{\partial W_2^{\left( 1\right) }}{\partial {I_{4,e}^{\left( 1\right) }}}\lambda _e^2\right] , \end{aligned}$$

where, the partial derivatives of the SEFs for the matrix and the fibres with respect to their strain invariants are as follows:

18 $$\begin{aligned} \frac{\partial W_0}{\partial {I_1}}= & c_1; \quad \frac{\partial W_1^{\left( 1\right) }}{\partial {I_4^{\left( 1\right) }}} = \left[ I_4^{\left( 1\right) }-1\right] k_1^{\left( 1\right) } e^{ k_2 [I_4^{\left( 1\right) }-1]^2}; \end{aligned}$$

19 $$\begin{aligned} \frac{\partial W_2^{\left( 1\right) }}{\partial {I_{4,e}^{\left( 1\right) }}}= & 2C_{2}^{\left( 1\right) }[I_{4,e}^{\left( 1\right) } - 1] + 3C_{3}^{\left( 1\right) }[I_{4,e}^{\left( 1\right) } - 1]^2 + 4C_{4}^{\left( 1\right) }[I_{4,e}^{\left( 1\right) } - 1]^3. \end{aligned}$$

From the definitions found in Eqs. (4) and (10), and for uniaxial tension, the invariants can be written in terms of global stretch, $\lambda$, and the stretch component $\lambda _e$ as follows:

20 $$\begin{aligned} I_1 = \lambda ^2 + 2\lambda ^{-1}; \quad I^{(1)}_{4}= \lambda ^2; \quad I^{(2)}_{4}= \lambda ^{-1}; \quad I^{(1)}_{4,e} = \lambda _e^2; \quad I^{(2)}_{4,e} = \lambda _e^{-1}. \end{aligned}$$

These are inserted, along with the partial derivatives, into Eq. (17) to obtain the expression for the one-dimensional first PK stress in terms of stretch. The same is then done for the circumferential direction, i.e. when $\varvec{N}^{(2)}$ is parallel to the axis of loading. The evolution of the elastic deformation found in Eq. (11) for the uniaxial tensile test condition can be written in its one-dimensional form in terms of $\lambda _e$ as follows:

21 $$\begin{aligned} \dot{\lambda }_e=\lambda _e\frac{\dot{\lambda }}{\lambda }-\frac{4}{3\eta _0}\frac{\partial W_2}{\partial I_{4,e}}\lambda _e^5 \end{aligned}$$

### Parameter identification and model validation

**Fig. 15:**
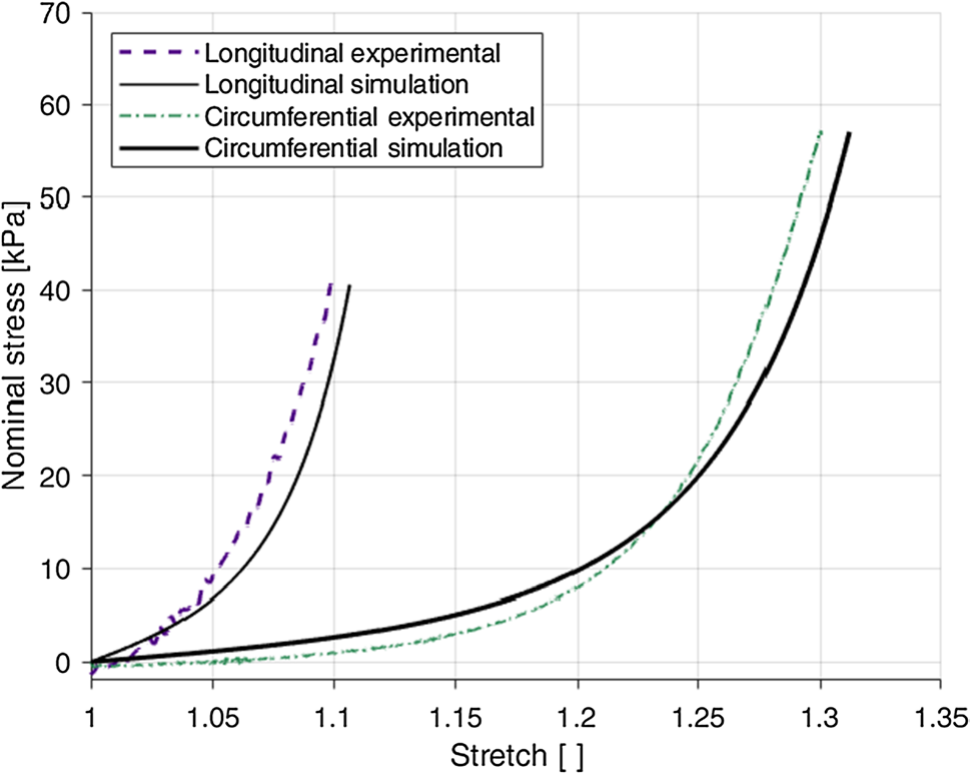
Identification of the hyperelastic parameters from the 1% s$^{-1}$ experimental results using the loading path of the second cycle of the final full stretch level of the longitudinal and circumferential directions

The first step of parameter identification was to identify the hyperelastic parameters by isolating the hyperelastic portion of the model from the softening function and viscoelasticity. This means that the purely hyperelastic form of the first PK stress equations were used to find these parameters. They were obtained by simulating the loading path of the second cycle of the final full stretch level of the 1%$s^{-1}$ experimental results of each direction, as can be seen in Fig. 15, as this provides the approximate preconditioned behaviour of the layer. First, the $c_1$ parameter was identified using the initial portion of the curve for the longitudinal and circumferential directions, and was fit simultaneously using a manual slider. Then, the $k_1^{(i)}$ and $k_2^{(i)}$ parameters were identified separately for each direction, also by means of a manual slider. Figure 15 shows the simulation of the hyperelastic portion of the model, which proves a good fit with the identified parameters: $c_1=0.86$kPa, $k_1^{(1)}=23.5$kPa, $k_1^{(2)}=3.98$kPa, $k_2^{(1)}=24.4$ and $k_2^{(2)}=3.85$. Note that these parameters will be kept frozen during the identification of the damage and viscous parameters.

**Fig. 16:**
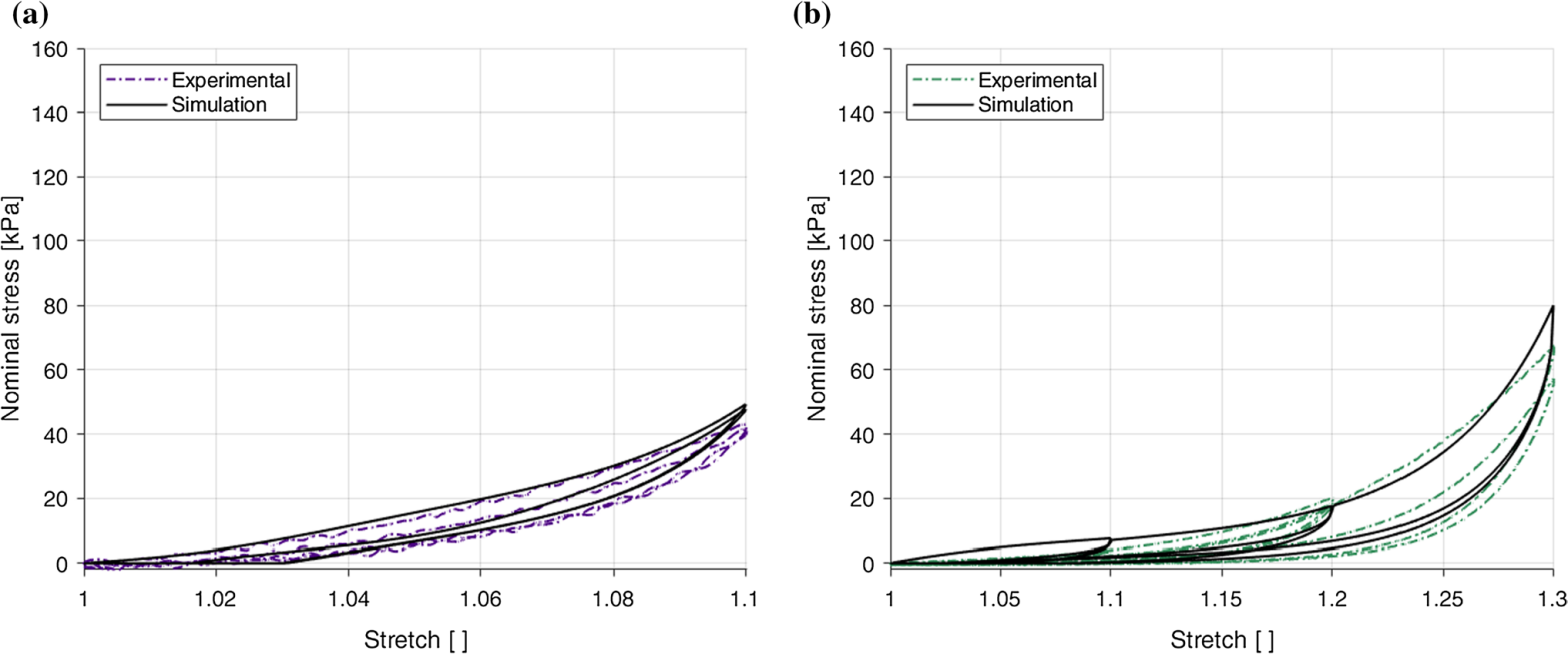
Parameter identification and modelling of the cyclic behaviour of the embalmed mucosa–submucosa layer for the 1%$s^{-1}$ experimental results in the longitudinal direction (**a**) and the circumferential direction (**b**)

**Table 5:** A complete set of material parameter values for the visco-anisotropic damage model identified in a modularised way

	$c_1 (\text {kPa})$	$k_1 (\text {kPa})$	$k_2 (-)$	$\eta _m (-)$	$\beta (-)$	$\eta _{0}$	$C_{2} (\text {kPa})$	$C_{3} (\text {kPa})$	$C_{4} (\text {kPa})$
$N^{(1)}$	0.86	23.5	24.4	0.09	0.66	824	5.50	0.01	1846
$N^{(2)}$		3.98	3.85	0.89	0.31	371	12.8	0.01	65.4

**Fig. 17:**
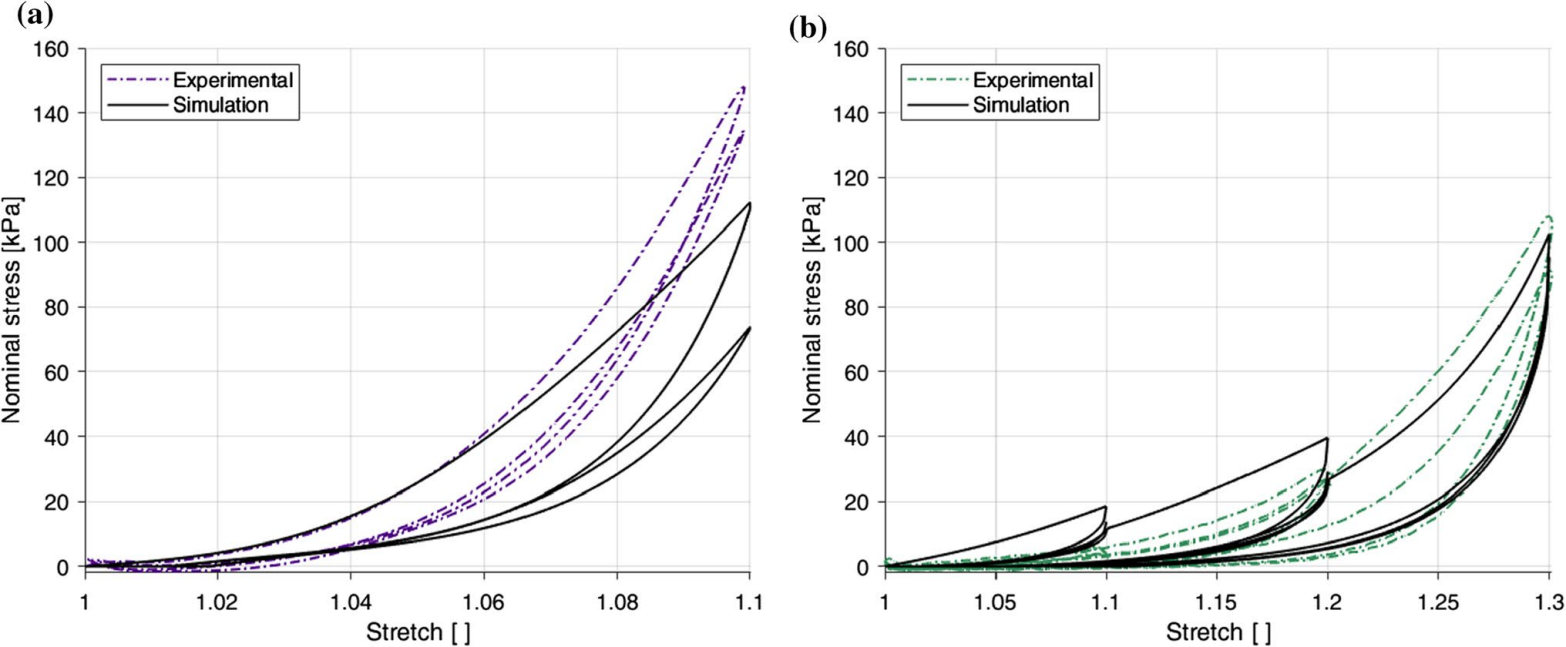
Parameter validation and modelling of the cyclic behaviour of the embalmed mucosa–submucosa layer for the 10%$s^{-1}$ experimental results in the longitudinal direction (**a**) and the circumferential direction (**b**)

The identification of the stress-softening and viscous parameters was conducted using the cyclic 1% s$^{-1}$ results. For this, the parameters were identified for each direction separately using the lsqcurvefit function in MATLAB. These fittings can be seen in Fig. 16, in which the model provides a good simulation of the behaviour in the circumferential direction and a very good simulation of the stress-stretch response in the longitudinal direction. All parameter values (hyperelastic, damage, and viscous) can be found in Table 5. The next step was to validate the model with a completely new set of data that had not been used in the parameter identification process. For this, the 10% s$^{-1}$ cyclic results were predicted for both directions. The results of the model validation are depicted in Fig. 17. The longitudinal direction results, shown in Fig. 17a, predict very well the behaviour of this direction up until approximately 1.045 stretch. However, past this point it can be seen that the model underestimates the change in stiffness experienced at the higher strain rate, while the hysteresis and difference between the two loading-unloading paths are overestimated compared to the experimental data. Overall, the model performs fairly well in capturing the behaviour of the longitudinal direction. The circumferential direction validation results, as seen in Fig. 17b, are found to overestimate the stiffness and hysteresis seen in the first two stretch levels of the stress-stretch response, but provide a very good prediction of the strain rate dependency at higher stretches, resembling well the non-linearity and hysteresis of the experimental results. The simulations for the circumferential direction, however, underestimate the hysteresis of the second cycle for each stretch level. That is to say, the model does not ideally capture the estimated preconditioned behaviour of the layer in the circumferential direction.

## Discussion

This study provides a unique insight into the anisotropic, layer-specific visco-hyperelastic behaviour of the human oesophagus through cyclic experimentation of the mucosa–submucosa layer, conducted under uniaxial tension and at two different strain rates. The mucosa–submucosa of three human oesophagi fixed in formalin were subjected to increasing stretch level cyclic tests in both the longitudinal and circumferential directions. The tests revealed hysteresis and permanent deformations of the layer, as well anisotropy and stress-softening; phenomena expected due to the prevalence of time-, history- and direction-dependent behaviour within soft tissues. The cyclic behaviour was simulated using an anisotropic matrix-fibre model, of which the fibre orientations were established through the histological findings of the study.

When considering the loading paths of each first cycle, the overall stiffness of the mucosa–submucosa layer was found to be greater in the longitudinal direction than in the circumferential direction, with the $k_1$ hyperelastic parameters of the model confirming this. This can be related to the histological results, where the greater proportion of collagen residing in the longitudinal direction of the mucosa–submucosa can be linked to the greater stiffness seen in this direction, as collagen is known to be a predominant component contributing to the tensile strength of soft tissues (Aziz et al. 2016). This finding is in line with similar animal studies investigating the layer-dependent, anisotropic properties of the oesophagus (Sommer et al. 2013; Stavropoulou et al. 2012).

Non-linearity of oesophageal tissue has been related to its physiological function wherein the wall displays compliance at low strains to accommodate for the swallowing process, but stiffens at high strains in order to prevent over-dilatation (Mir et al. 2016). The muscular layer is said to account for the resistance seen at low intraluminal pressures, while the mucosa–submucosa is seen to rapidly increase in stiffness only when the outer diameter is stretched to around double its original size (Goyal et al. 1971; Gregersen 2003). The results of this study show a representation of this mucosal behaviour in the circumferential direction, wherein low stresses are seen at low stretches, particularly for the 1% s$^{-1}$ results, with the direction stiffening exponentially as the stretch level increases. However, stiffening in the study occurs much earlier than when the samples have been stretched to double their original length. This could be due to the effects of embalming on the tissue, as the process has been found to influence the stiffness of soft tissues when compared with their fresh counterparts. When considering studies investigating these embalming effects, the exact influence on soft tissues is inconclusive. Formalin is known to add cross-links to collagen (Fessel et al. 2011), which may explain the increase in tissue stiffness caused by embalming found by Hohmann et al. (2019) and the early stiffening of the circumferential direction in this study. However, Girard et al. (2019), who studied the effects of preservation processes on the cyclic behaviour of the human bile duct, found that the stiffness of embalmed tissue decreased, and the permanent set in the longitudinal direction increased, when compared to the fresh counterpart (Girard et al. 2019). In addition, when compared with the circumferential stretch of the oesophagus established by Takeda et al. (2002), as referenced in Sect. 2.6, the results of this study reveal the circumferential direction of the mucosa–submucosa to rupture well before reaching the upper limit of 1.7 stretch. Despite Takeda et al. (2002) investigating the intact oesophageal wall, the rupture stretch of the circumferential direction here is much lower than expected. It is anticipated, therefore, that the embalming process of the current study has resulted in changes to both the damage and stiffness of the samples; the exact direction of influence of which, however, is currently unknown. It is also useful to note that the Young’s moduli of both directions in this study may be greater than those expected for younger tissues due to the high ages of the patients tested and the effects of ageing on the mechanical properties of oesophageal tissue (Vanags et al. 2003). In spite of these factors, greater compliance at lower stretches was seen in the circumferential direction compared to the longitudinal direction, which is thought to allow for the passage of varying sizes of fluid bolus. The role of the longitudinal muscle fibres during peristalsis is to enact local shortening, so the greater stiffness seen in this direction, even in the mucosa–submucosa layer, is thought to support this function.

A variety of viscoelastic behaviours were observed within the cyclic experimental results. Stress-softening is defined as a history-dependent damage mechanism whose effect depends only on the previous maximum stretch of the material. This phenomenon was seen for all trials, wherein the stiffness of the second cycle for a single stretch level was much lower than that for the first cycle. Hysteresis was also found to decrease for the second cycle when compared to the first, with the second cycle representing approximately the behaviour of the tissue if the sample had been preconditioned. In terms of hysteresis, the dissipated energy was greater in the circumferential direction compared to the longitudinal direction. As outlined in the Introduction, Liao et al. (2009) established that the majority of softening of guinea pig oesophagi was due to irreversible structural changes dependent on the previous maximum stretch, and only partly due to the time-dependent softening wherein the viscoelasticity of the tissue causes reversible softening dependent on the strain rate. This cannot be entirely assumed for the human oesophagus, however strain rate-dependent behaviour was less prominent in the circumferential direction compared to the longitudinal, suggesting that stretch history may have more of a contribution to the softening in the circumferential direction. In the longitudinal direction, the difference between the two cycles was greater at the higher strain rate and there was also a more pronounced strain rate dependency, implying that time-dependent softening has more of an impact in this direction. This corresponds to the histological results in that the greater viscoelasticity of the longitudinal direction could be attributed to the preferential alignment of collagen within the layer (Li et al. 2005).

Permanent deformations were seen for all test conditions. Despite only considering one stretch level in the longitudinal direction, the permanent set in this direction was greater at the faster strain rate. This was also the case for the circumferential direction, where a clear trend of permanent set is more visible due to the ability to compare across several stretch levels. When comparing across a single strain rate, there were greater permanent deformations in the circumferential direction than the longitudinal direction. This suggests that greater irreversible structural changes occur in the circumferential direction than in the longitudinal direction. For both strain rates, the longitudinal samples ruptured before the circumferential samples. It is therefore hypothesised that, along with the greater permanent set in the circumferential direction, the mucosa–submucosa layer allows more easily for the breakage of cross-links between molecules in the circumferential direction compared to the longitudinal, resulting in a greater resistance to complete fracture. In other words, the damage of the microstructure predominately in the circumferential direction could lead to the prevention of premature macrostructure fractures, more important in the circumferential direction due to the variable nature of bolus sizes. However, analysis of the layer’s microstructure during and after testing is required for confirmation of this.

The viscoelastic behaviour was captured relatively well by the matrix-fibre model which incorporated the anisotropy, stress-softening and strain rate dependency observed in the experimental results. Overall, the model provided a good simulation of the behaviour in both directions at 1%$s^{-1}$, while the prediction of the 10%$s^{-1}$ results was more accurate in terms of stiffness for the circumferential direction compared to the longitudinal direction. It should be noted that the lower strain rate of 1%$s^{-1}$ may be too high to sufficiently distinguish the stress-softening from the viscoelastic behaviour when modelling the tissue response. Therefore, for the mechanical tests, an increase in the number of strain rates tested, e.g. to include 0.1%$s^{-1}$, would allow for a more comprehensive characterisation of the mucosa–submucosa’s quasi-static response and strain rate-dependent behaviour.

## Conclusion

This study investigated the layer-specific mechanical behaviour of the human oesophagus through experimentation of the embalmed mucosa–submucosa layer, considering directionality by testing in both the longitudinal and circumferential directions. The results revealed the mucosa–submucosa to exhibit highly anisotropic, visco-hyperelastic behaviour with stress-softening; the data of which has been used to numerically model, with relative success, the response of the tissue. The longitudinal direction was found to be consistently stiffer than the circumferential direction, which is in line with similar oesophageal animal studies (Sommer et al. 2013; Stavropoulou et al. 2012; Yang et al. 2004). Not known to have been previously studied using human tissue, the results presented here provide a unique insight into the layer-specific cyclic behaviour of the human oesophagus, while also offering an individual formulation of a visco-hyperelastic constitutive model able to capture the anisotropy, hyperelasticity, viscoelasticity, permanent set, hysteresis, stress-softening and strain rate-dependent behaviour of the tissue layer. Upon lifting of the COVID-19 restrictions, the layer-specific properties of the fresh human oesophagus will be established and compared with the results of this study, along with those of the embalmed muscularis propria layer (Durcan et al. 2022), allowing for a discussion of the effects of embalming on the material behaviour of the oesophagus, and on soft human tissues in general. Further to this, the fresh tissue results will be constitutively modelled to contribute more physiologically-relevant parameters, with a Finite Element implementation of this in the plans for future work.
